# In Memoriam: William Weston

**DOI:** 10.2196/46576

**Published:** 2023-08-09

**Authors:** Robert P Dellavalle, Anna L Bruckner, Lela A Lee

**Affiliations:** 1 University of Colorado Anschutz School of Medicine Aurora, CO United States; 2 American Board of Dermatology Denver, CO United States

**Keywords:** pediatric dermatology, neonatal lupus

Dr William “Bill” Weston, born on August 13, 1938, in Grand Rapids, MN, passed away on November 13, 2022, at the age of 84 years in Denver, CO. His family relocated to Richmond, WA, when he was a young child. He graduated from Whitman College in Walla Walla, WA, in 1960 with a bachelor’s degree in chemistry. He received a Bachelor of Medical Sciences degree from the University of South Dakota, where he studied from 1961 to 1963 and met the love of his life, his wife Janet Atkinson Weston, MD, who also trained in pediatrics.

He pursued medicine at the University of Colorado, where he received his medical degree in 1965, completed an internship, and started a residency in pediatrics under the mentorship of C Henry Kempe, MD. He completed his pediatric residency at the University of California San Francisco. After serving in the US Army, he returned to Colorado to complete another residency in dermatology (1970-72) and a fellowship in immunodermatology (1972-73).

Dr Weston served as the chair of the Department of Dermatology at the University of Colorado School of Medicine for over 25 years from 1976 to 2001 and as the section head of Pediatric Dermatology for 33 years from 1972 to 2005. He was also on staff at the Fitzsimons Army Medical Center (1972-96), the Denver Veterans Administration Medical Center (1976-2006), and the Denver Health Medical Center (1976-2007).

**Figure 1:**
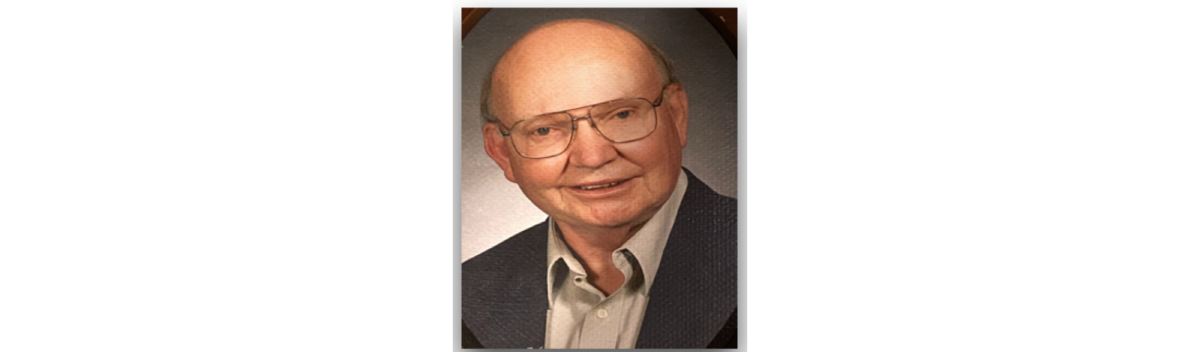
This is an undated photo of Dr William Weston.

Dr Weston’s impact on the field of pediatric dermatology was remarkable. He authored a leading textbook in the field [1] and trained more than 30 practicing pediatric dermatologists globally. He was a founding father of the field and among the first to describe the signs of neonatal lupus [2].

Throughout his career, Dr Weston received numerous accolades, including the University of Colorado School of Medicine–Florence Rena Sabin Award in 2000, the American Academy of Pediatrics–Alvin H Jacobs Award in 2003, and the University of Colorado Medal in 2022. He published over 350 articles during his career.

Dr Weston was an accomplished clinician, educator, scholar, leader, mentor, and friend. His research mentorship was instrumental in launching the careers of many academic dermatologists, and his knowledge was encyclopedic. Dr Weston had a genuine love of children and a unique way of describing them. He was also an avid baseball fan and traveled to every Major League Baseball field in the country with his son to watch a game on each team’s home field.

Dr Weston was an unassuming man who approached each patient with humility and compassion. His legacy as a humble and generous leader and role model will always guide the relationships of his trainees and colleagues. We are grateful to have received so many gifts from Dr Weston, and we owe our success to his early involvement in our careers.

We celebrate Dr Weston’s life with deep gratitude. Thank you, Dr Weston, for investing in us and in every other trainee who had the privilege of learning from you. Thank you for sharing your talents, time, and energy with the world so freely. Your memory will always be cherished, and your impact will be felt for generations to come.
